# Microvascular density of regenerative nodule to small hepatocellular carcinoma by automated analysis using CD105 and CD34 immunoexpression

**DOI:** 10.1186/1471-2407-14-72

**Published:** 2014-02-07

**Authors:** Juliana Passos Paschoal, Vagner Bernardo, Nathalie Henriques Silva Canedo, Osmar Damasceno Ribeiro, Adriana Caroli-Bottino, Vera Lucia Pannain

**Affiliations:** 1 Department of Pathology/University Hospital, Federal University of Rio de Janeiro, Rua Prof. Rodolpho Paulo Rocco, 255, Cidade Universitária, CEP: 21941-913 Rio de Janeiro, RJ, Brazil; 2 National Cancer Institute [Molecular Carcinogenesis Program – CPQ/INCA] Rua André Cavalcante, 37, Centro, CEP: 20231-050 Rio de Janeiro, RJ, Brazil

**Keywords:** Microvascular density, Regenerative nodule, Dysplastic nodule, Hepatocellular carcinoma, CD105, CD34

## Abstract

**Background:**

Angiogenesis is a proliferative process resulting in the development of new blood vessels from existing endothelial cells and is considered crucial for tumor growth and metastasis. Tumor angiogenesis can be quantified by microvascular density (MVD), which is evaluated in highly vascularized tumor areas (hot spots) by immunohistochemical assays using CD34 and CD31 pan-endothelial antibodies. More recently, CD105 has been successfully used for some tumor types because it could discriminate neovascularization. The expression of CD34 and CD105 in hepatocellular carcinomas (HCC) and hepatic precancerous lesions has been reported—although the results for CD105 are controversial—but to the best our knowledge, CD105 has not been previously investigated in dysplastic nodules (DN). We investigated and compared MVD-CD34 and MVD-CD105 immunoexpression in tissues containing different stages of hepatocarcinogenesis, including DN.

**Methods:**

A total of 31 regenerative nodules (RN), 26 DN and 25 small HCC from explants were used for immunohistochemical tests with CD34 and CD105 antibodies. Antibody expression was quantified by computerized image analysis measurement of MVD, areas containing highly positive endothelial cells within the nodules.

**Results:**

The median MVD for CD34 was higher in HCC than in DN and RN (p < 0.01), and was higher in DN compared with RN (p = 0.033). In contrast, MVD with CD105 was higher in RN, and the difference was significant in RN and DN compared with HCC (p = 0.019 and p = 0.012, respectively). When MVD with CD34 and CD105 were compared within a single group, there was a significant predominance of CD105 in RN and DN (p < 0.01). In addition, MVD-C34 in HCC predominated compared with MVD-CD105, but the difference was not statistically significant (p = 0.128).

**Conclusions:**

This study identified a close relationship between CD105 and liver cirrhosis, and that CD34 antibody is a good endothelial marker for hepatic carcinogenesis. There was no difference between the use of CD105 and CD34 antibodies in preneoplastic lesions.

## Background

Angiogenesis is a proliferative process resulting in the development of new blood vessels from existing endothelial cells and occurs during reproduction, development and wound repair. The angiogenic process includes cell migration, proliferation, microvascular differentiation, extracellular matrix degradation and structural reorganization [1]. Folkman’s hypothesis that tumor growth is angiogenesis-dependent was confirmed by biological, pharmacological and genetic evidence [2]. Endothelial progenitor cells from bone marrow are recruited to vascular bed tumors and contribute to tumor growth [3].

For years it was thought that the formation of new blood vessels occurred after the cells acquired a malignant phenotype; however, experimental and clinical evidence has demonstrated that angiogenesis is increased in some premalignant lesions in cervical, lung and in adenoma-carcinoma colon cancer sequence [4-6]. It was also observed in the evolution of MGUS (Monoclonal Gammopathy of Undetermined Significance) [7].

Tumor angiogenesis is usually quantified as microvascular density (MVD) [8]. MVD is evaluated in highly vascularized tumor areas (hot spots) by immunohistochemical assays using pan-endothelial antibodies (CD34, CD31 and von Willebrand factor). It is assumed that angiogenic activity is associated with the development and progression of some solid tumors and has an important prognostic value [9-12]. Recently, evidence demonstrated that another endothelial marker, endoglin (CD105), is overexpressed in active angiogenesis and might be a useful marker of neoangiogenesis, because it can discriminate immature vessels from the mature and established vessels [13,14]. Furthermore, endoglin is undetectable or weakly expressed in the endothelium of normal tissues [15]. In liver it was observed in very few endothelium cells in the vicinity of veins [15]. Endoglin is a transmembrane accessory receptor of the transforming growth factor beta receptor system [16] expressed mainly in vascular endothelial cells and is a diagnostic and therapeutic molecular target for cancer. CD105 expression has been detected by immunohistochemistry for the evaluation of angiogenesis in premalignant and malignant lesions. It is considered more neoangiogenesis-specific than pan-endothelial CD34 and CD31 antibodies and might have a more significant prognostic value for some cancers [14,17,18]. The role of angiogenesis in chronic liver disease, liver premalignant lesions and liver cancer has also been studied using pan-endothelial antibodies [19]. However, studies of endoglin and angiogenesis have been controversial [20,21], and no studies have reported the association between endoglin and liver premalignant lesions. The purpose of this study was to determine and compare MVD with CD105 and CD34 antibodies in small hepatocellular carcinomas (HCC), regenerative and dysplastic liver nodules.

## Methods

This study used samples from 31 regenerative nodules (RN), 26 dysplastic nodules (DN) and 25 small HCC from the Department of Pathology/University Hospital, UFRJ. The samples were obtained from 28 patient liver cirrhotic explants who underwent surgery between 2000 and 2007. The explants specimens were 10% buffered-formalin fixed and paraffin-embedded using standard histology methodology to ensure the viability of tissues for further immunohistochemical studies. Lesions were histologically classified according to IWP guidelines [22]. Patients consisted of 16 males and 12 females with a mean age of 55 years. Hepatitis C virus (HCV) infection was the main etiological factor of liver cirrhosis (82.1%), followed by hepatitis B virus (7.1%), alcohol, and biliary and cryptogenic etiologies (3.6%). This study was approved by the local ethics committee (CEP:237/07).

### Immunohistochemistry

Immunohistochemical staining was performed for CD105 and CD34 antibodies in 4 μm thick tissue sections from paraffin blocks. The commercially available monoclonal anti-CD34 (1:50 dilution, M7165, clone QBEnd-10; Dako, A/S DK) and monoclonal anti-CD105 (1:30 dilution, M3527, clone SN6h 1; Dako, Carpenteria, CA, USA) were used. The Universal LSAB™2 Kit/HRP, Rabbit/Mouse-K0675 (Dako, Carpenteria, CA, USA) and Novolink (Novocastra, Newcastle, UK) RE7140-CE DAB detection systems were used for anti-CD34 and anti-CD105, respectively. Negative controls consisted of the reaction performed without primary antibodies and positive controls consisted of placenta and granulation tissue for CD34 and CD105, respectively.

### Microvascular density

Microvascular quantification was performed inside the nodules, not in fibrous septum or capsule, using automated analysis of images as previously described in a pioneer reproducibility-tested study [23]. Briefly, the sections were scanned at × 100 magnification (×10 objective and ocular lens) for selection of the most immunopositive CD34 and CD105 sinusoidal areas (hot spots). Subsequently, two to five fields were captured from each nodule, depending on the nodule size. The images were captured by Qcolor 5 video camera (Olympus) attached to Olympus BX-51 microscope, using × 200 magnification (×20 objective and × 10 ocular lens). The illumination was kept constant during all image capture. The area measured in each image was 692.76 × 519.56 μm. The MVD areas were quantitatively measured using Image-Pro plus 6.2.1 software (Media Cybernetics, Silver Spring, EUA). The final MVD of each sample was calculated by the ratio of the sum of the immunopositive areas and the sum of the total area.

### Statistical analysis

Fisher’s exact test and chi-square were used to compare the immunostaining results between the antibodies in different lesions (RN, DN and HCC). Tests were considered significant when p values were < 0.05. Data normality of MVD was verified using the Kolmogorov-Smirnov test. Normality was rejected for CD34 antibody (p = 0.000) and CD105 antibody (p = 0.001). The nonparametric Kruskal-Wallis test was used to compare the groups to verify similar distribution. When the distributions were different, the Mann-Whitney *U-*test was applied.

## Results

**Figure 1:**
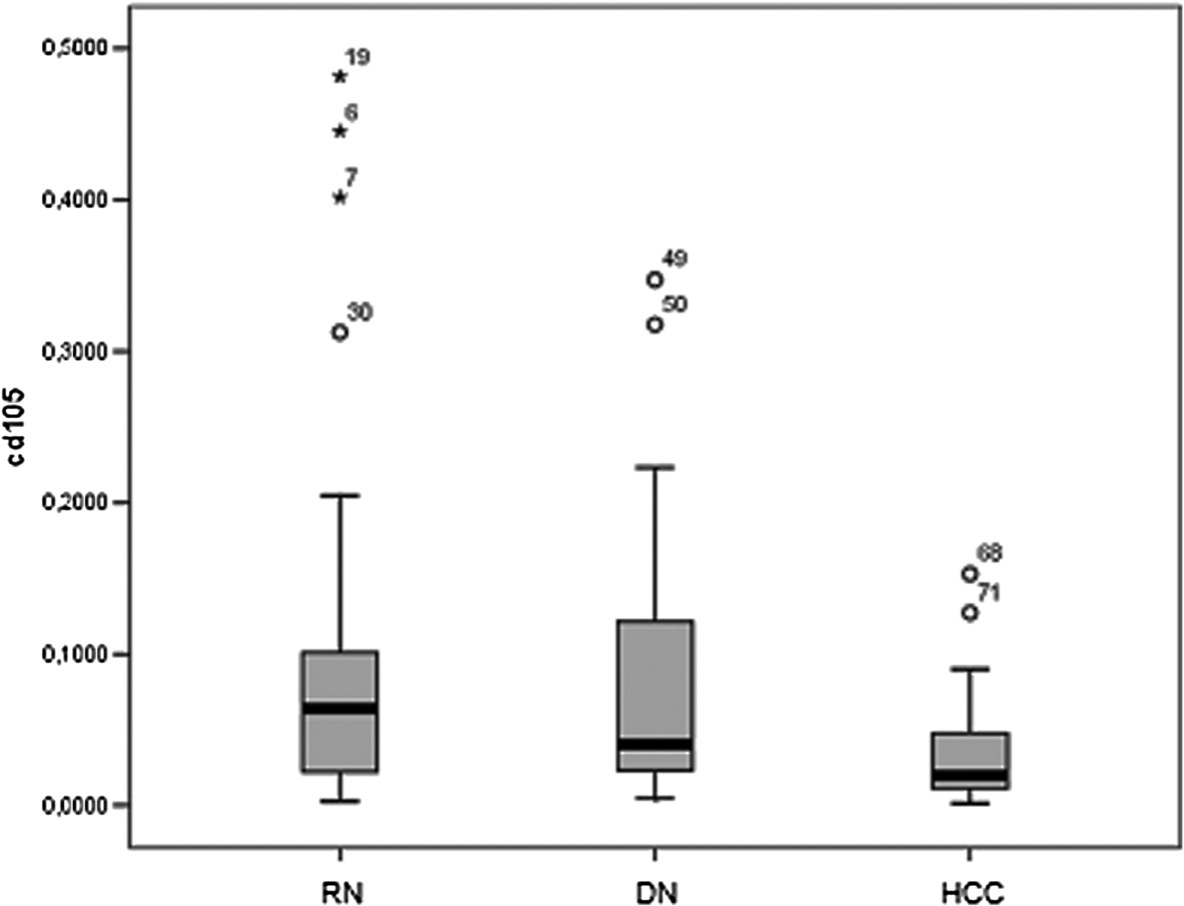
**MVD expression ratio for CD105 antibody according to diagnostic group.** Y axis represents MVD for each group. RN = regenerative nodules; ND = dysplastic nodules; HCC = hepatocellular carcinoma.

**Figure 2:**
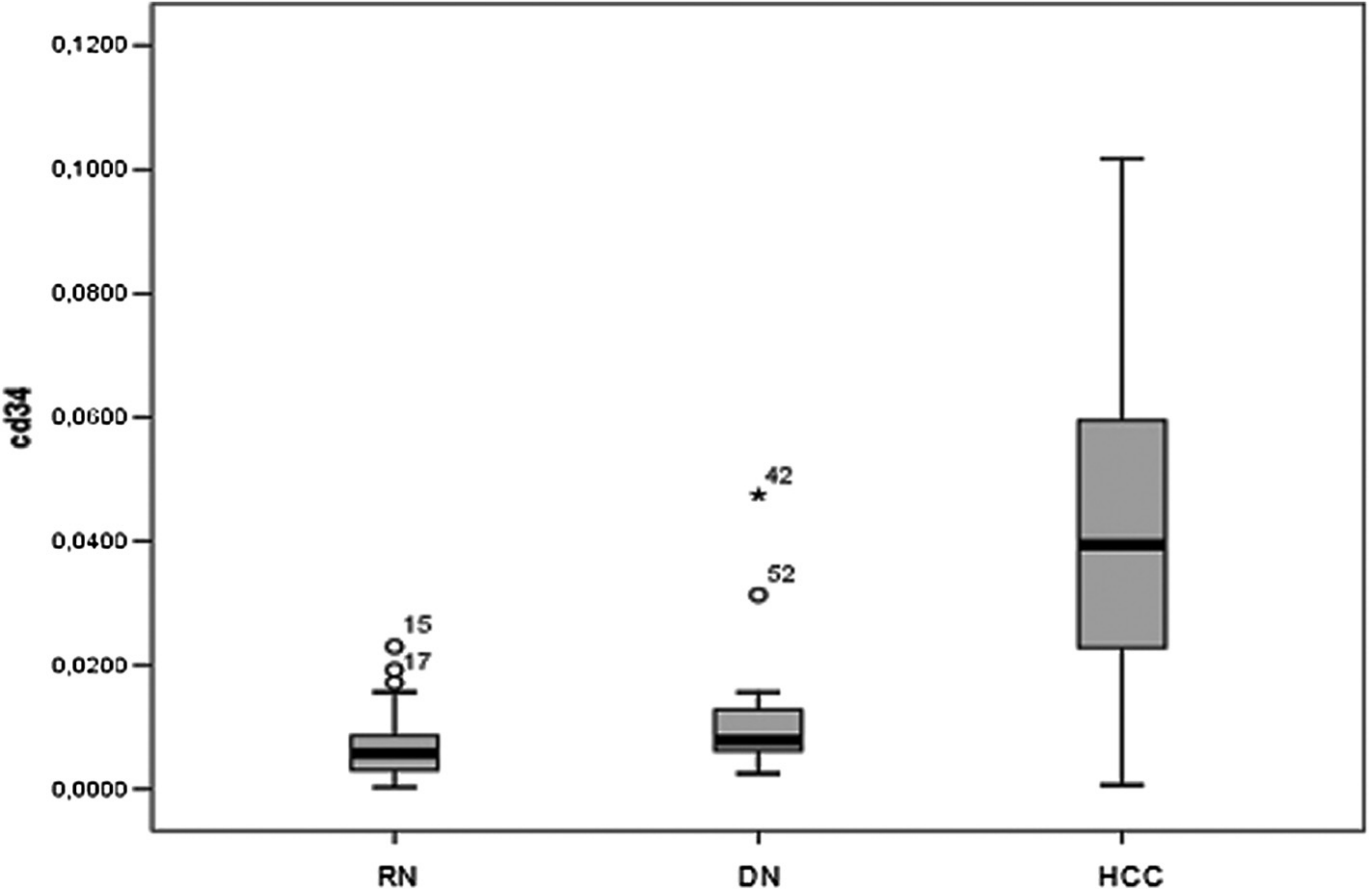
**MVD expression ratio for CD34 antibody according to diagnostic group.** Y axis represents MVD for each group. RN = regenerative nodules; ND = dysplastic nodules; HCC = hepatocellular carcinoma.

**Figure 3:**
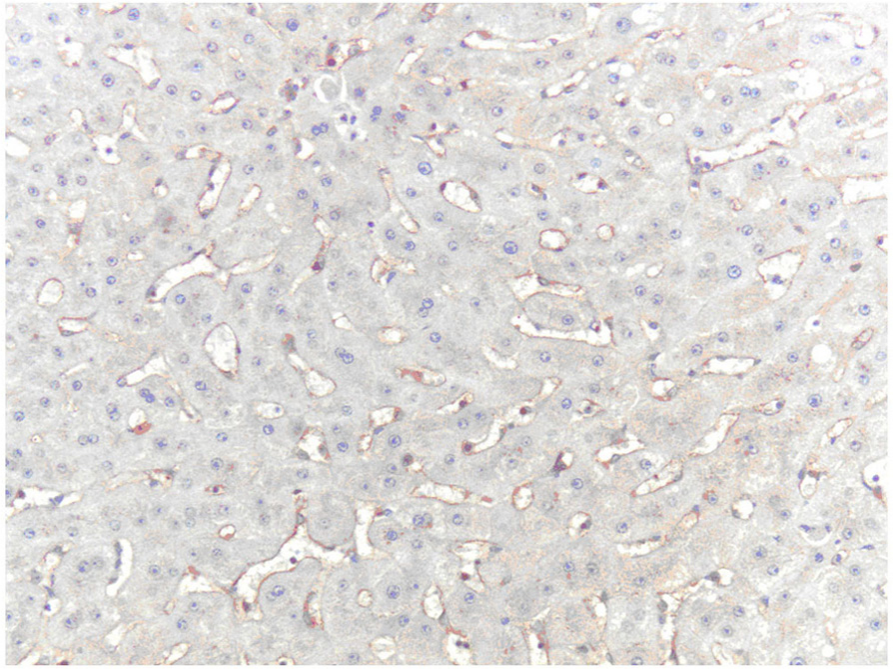
**CD105 immunoexpression in sinusoidal cells of RN (hot spot area).** Original magnification, x400.

**Figure 4:**
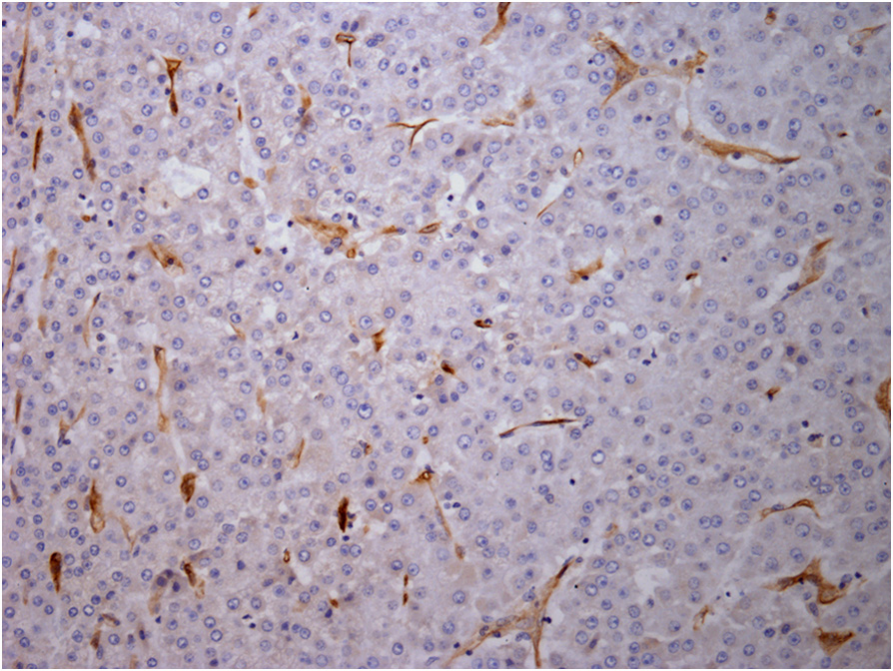
**CD105 immunoexpression in microvessels of HCC (hot spot area).** Original magnification, x400.

**Figure 5:**
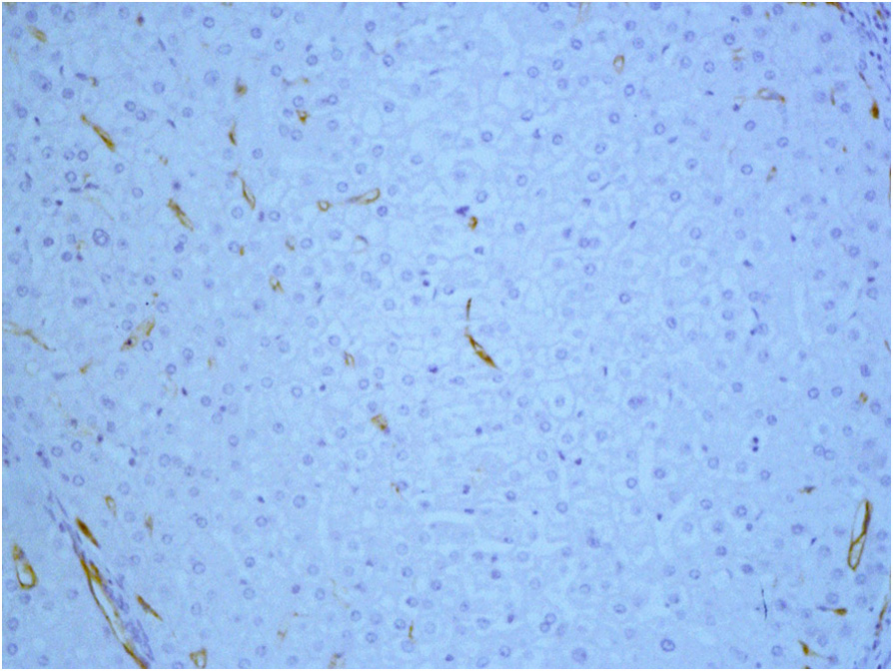
**CD34 immunoexpression in sinusoidal cells of RN (hot spot area).** Original magnification, x400.

**Figure 6:**
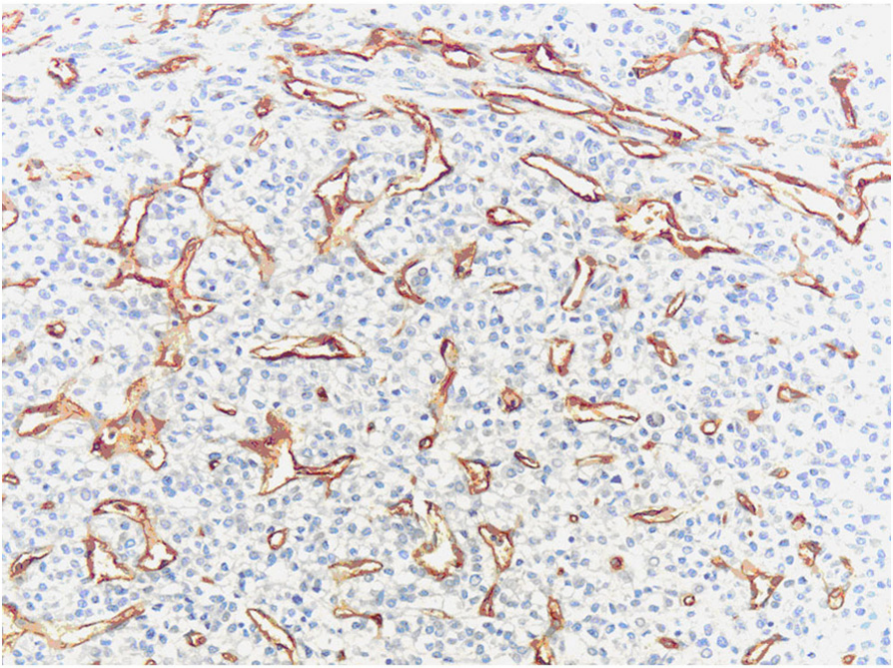
**CD34 immunoexpression in microvessels of HCC (hot spot area).** Original magnification, x400.

The CD105 and CD34 endothelium markers were observed in all types of hepatocellular nodules, although with variable intensity (Tables 1 and 2). As shown in Figure 1, the MVD-CD105 score was significantly higher in RN than in DN and HCC (p = 0.02). In addition, the MVD-CD34 score increased from RN to HCC (Figure 2). The MVD-CD34 score was significantly higher in HCC than DN and RN p < 0.01 in both cases, as well as between DN and RN (p = 0.03). It was observed that when MVD-CD34 and MVD-CD105 scores were compared within a single group, the sinusoidal area stained by anti-CD105 was significantly greater than with anti-CD34 in RN and DN (p < 0.01). However, no significant association was found in HCC between anti-CD34 and anti-CD105 despite the higher CD34 score. Representative images of the immunohistochemical expression of CD105 and CD34 in RN and HCC are shown in Figures 3, 4, 5, and 6.

**Table 1:** CD105 MVD value in hepatocellular nodules

	**N**	**Mean**	**Inferior**	**Superior**	**Minimal value**	**Maximal value**	**Median**	**SD**
RN	31	0.103	0.054	0.151	0.003	0.481	0.064	0.132
DN	26	0.087	0.049	0.125	0.004	0.347	0.040	0.093
HCC	25	0.037	0.021	0.053	0.001	0.153	0.020	0.039

RN = regenerative nodule; DN = dysplastic nodule; HCC = hepatocellular carcinoma; SD = standard deviation.

CD105 MVD value - RN compared to DN and HCC: p = 0.02.

**Table 2:** CD34 MVD value in hepatocellular nodules

	**N**	**Mean**	**Inferior**	**Superior**	**Minimal value**	**Maximal value**	**Median**	**SD**
RN	31	0.007	0.005	0.009	0.000	0.023	0.006	0.006
DN	26	0.011	0.007	0.014	0.002	0.048	0.008	0.009
HCC	25	0.043	0.031	0.054	0.001	0.102	0.039	0.027

RN = regenerative nodule; DN = dysplastic nodule; HCC = hepatocellular carcinoma; SD = standard deviation.

CD34 MVD value - HCC compared to DN and RN: p < 0.01.

## Discussion

Angiogenesis studied by immunohistochemical methods has proven to be important for assessing prognosis in some neoplasias. Increased levels of antibodies to endoglin, CD31 and CD34 are associated with progression-free survival tumor grade and metastasis [11]. Angiogenesis in liver diseases is peculiar because immunophenotype changes of endothelial sinusoidal cells occur in cirrhosis, causing cells to express vascular markers not found in normal livers. They are also observed in precancerous lesions and are considered part of the hepatocarcinogenesis process [24,25].

Angiogenesis quantification (MVD) in tumors is performed by counting vessels stained with specific antibodies to evaluate a relationship between antibody marker and prognosis, allowing the selection of patients whose tumors may respond to antiangiogenic therapy. MVD may be influenced by the antibody used for quantification, as well as the number of fields counted and the area where the vessels are quantified. Weidner et al. [9] proposed evaluation of MVD in areas of high concentration of vessels (hot spots) in breast cancer. This method has since been performed for other tumor types [26,27]. However, automated analysis image to assess MVD is a better method than manual quantification because it evaluates larger areas of tumor, is easily reproducible, has higher accuracy and minimizes inter-observer variability [28].

In liver carcinogenesis, CD34 antibody is one of the most studied vascular markers. It is important for the prognostic evaluation of patients and also has diagnostic value, even though it does not directly reflect neoangiogenesis activity [12,27]. However, endoglin has been shown to be more specific than CD34 for angiogenesis determination, as its expression is detected mainly in new vessels, and consequently has a greater therapeutic potential. In this study, MVD-CD34 scores were significantly higher in small HCC than in DN and RN, which is in agreement with previous studies of advanced HCC [29]. These results demonstrate this new sinusoidal endothelium immunophenotype increases toward HCC and reaches the maximum score even in small HCC. Sinusoidal phenotype changes in these lesions could prevent endothelial rupture due to high pressure from arterial blood flow that occurs in HCC [15,30]. Conversely, endoglin MVD scores were significantly higher in cirrhosis than in HCC and DN, and were higher in DN compared with HCC. Our results are similar to previous studies that demonstrated higher endoglin expression in peritumoral tissue when compared with HCC [20]. The significant elevation of CD105 expression was also observed in the serum of patients with cirrhosis compared to healthy subjects [31]. In contrast, Yang et al. did not observe CD105 immunoexpression in non-neoplastic cells around tumors [32], although we speculate that the peritumoral tissue analyzed was not cirrhotic.

A possible explanation for the higher MVD-CD105 scores in cirrhosis is that endothelial sinusoidal cells acquire a neovessel immunophenotype due to endothelial cell hypoxia, inducible factors of hypoxia, persistent liver injury and hepatic regeneration, all of which contribute to increased CD105 expression [20,33]. There is evidence that endoglin is expressed in other cells including mesangial, fibroblasts and stellate cells in the liver although it is predominantly expressed in endothelial cells [34,35]. Clement et al. reported that stellate cells expressed endoglin and its upregulation was associated with progressive fibrosis in chronic hepatitis patients with HCV infection [35]. Considering these different possibilities, high MVD-CD105 levels in cirrhotic livers observed in this study might be due to CD105 immunostaining in sinusoidal endothelial cells and stellate cells. Therefore, before potential therapeutic antiangiogenic targeting with CD105 in HCC and cirrhosis patients, further studies investigating the function of endoglin in cirrhotic livers are required.

Another important finding in this study was higher MVD-CD105 scores in RN compared with DN and HCC, demonstrating CD105 expression decreases gradually from cirrhosis to DN to small HCC, opposite to CD34 expression. To our knowledge, this is the first endoglin study in DN. Regarding HCC, previous studies compared the expression of these markers and showed a predominance of CD34 compared with CD105, similar to that observed in the current study [12,20,29,32]. The use of endoglin antibodies are not recommended for routine diagnostic pathology of differentiated HCC to DN.

## Conclusions

This study demonstrated a close relationship between endoglin and liver cirrhosis, in contrast to CD34 antibody, which is a good endothelial marker of hepatic carcinogenesis. However, there is no difference between CD34 and CD105 antibodies in preneoplastic lesions.

## Abbreviations

MVD: Microvascular density; HCC: Hepatocellular carcinomas; RN: Regenerative nodules; DN: Dysplastic nodules; HCV: Hepatitis C virus.

## Competing interests

The authors declare that they have no competing interests.

## Authors’ contributions

JPP selected the cases, performed histological diagnosis and automated immunohistochemistry analysis, interpreted the results and helped to draft the manuscript. VB performed automated immunohistochemistry analysis. NHSC coordinated the immunohistochemistry study and reviewed the English language of the manuscript. ODR performed analysis and interpretation of the data. ACB performed histological diagnosis, and reviewed and submitted the manuscript for publication. VLP designed the study, performed histological diagnosis, interpreted the results and drafted the manuscript. All authors read and approved the final manuscript.

## Pre-publication history

The pre-publication history for this paper can be accessed here:

http://www.biomedcentral.com/1471-2407/14/72/prepub
